# Association of Vitamin D and Reproductive Hormones With Semen Parameters in Infertile Men

**DOI:** 10.7759/cureus.14511

**Published:** 2021-04-15

**Authors:** Sangeeta Kumari, Kalpana Singh, Shubhanti Kumari, Huma Nishat, Bhawana Tiwary

**Affiliations:** 1 Reproductive Medicine, Indira Gandhi Institute of Medical Sciences (IGIMS), Patna, IND

**Keywords:** vitamin d, male infertility, semen parameters, sex hormones, testosterone (tt)

## Abstract

Background

Hypovitaminosis D has been linked with poor semen parameters and endocrinological factors in male infertility. This study aimed to analyze the association of serum vitamin D levels and reproductive hormones such as follicle-stimulating hormone (FSH), luteinizing hormone (LH), and total testosterone (TT) with the semen parameters in a cohort of infertile men.

Methodology

In this study, 224 infertile men (aged 18-45 years) were recruited after applying inclusion and exclusion criteria. Semen analysis was performed as per the 2010 World Health Organization (WHO) criteria. The patients were divided into two groups on the basis of semen parameters: normozoospermic men and men with one or more altered semen parameters as per the WHO 2010 guidelines for all the analysis. Vitamin D and hormone levels were evaluated by quantitative competitive immunoassay by chemiluminescent microparticle immunoassay technology with flexible assay protocols (Architect, Abbott Diagnostics, Lake Forest, IL, USA). The patients were further divided into three groups on the basis of vitamin D levels: Sufficient (>20 ng/mL), insufficient (12-20 ng/mL), and deficient (<12 ng/mL). These groups were compared for different semen and hormonal parameters.

Results

Out of the 224 infertile men included, 124 were normozoospermic while 100 patients had one or more altered semen parameters. The median age of the participants was 30 years (minimum = 18 years, maximum = 45 years). The serum vitamin D and TT levels were significantly lower (p < 0.0001) and FSH (p = 0.001) and LH levels (p < 0.0001) were significantly higher in those with one or more altered semen parameters compared to the normozoospermic men. The sperm concentration, total motility, linear progressive motility, percentage normal morphology, and serum TT levels were significantly lower in the patients with serum vitamin D levels of <12 ng/mL in both the normozoospermic men and those with one or more altered semen parameters compared to patients with higher vitamin D levels. Semen parameters such as sperm concentration, total motility, linear progressive motility, and morphology were positively correlated with the levels of serum vitamin D and TT.

Conclusions

Vitamin D deficiency was examined in a large proportion of infertile men. Serum vitamin D and TT levels were positively associated with semen parameters evident from lower levels of vitamin D and TT in men with altered semen parameters compared to normozoospermic men. However, further comprehensive studies with larger sample sizes should be conducted to further validate the role of vitamin D in male infertility by checking the effect of vitamin D supplementation on semen parameters.

## Introduction

Vitamin D deficiency has become a global health problem. Several factors such as modern lifestyles (tendency to remain indoors frequently), food habits, and milk allergies have resulted in the increased prevalence of vitamin D deficiency making it a global public health concern [1]. Vitamin D3 is a key player in several important physiological and biochemical processes; however, it is primarily considered to be the regulator of calcium and phosphorus equilibrium and is implicated in bone health [2]. It is also involved in several other important processes such as fat metabolism [3], thyroid function [4], innate immunity [5], cardiovascular function [6], central nervous system [7], and the reproductive system [8]. As per reports, the prevalence of vitamin D deficiency ranges 30-93% in tropical countries where there is adequate sun exposure [1]. There is a high prevalence of Vitamin D deficiency throughout India. Vitamin D deficiency is prevalent at 70-100% in the general population in India. This high prevalence can be attributed to the rare fortification of food items, especially dairy products in India. Vitamin D deficiency is linked to the high prevalence of rickets, osteoporosis, cardiovascular diseases, diabetes, cancer, and infections such as tuberculosis in India [9].

Largely, the evaluation and treatment of male infertility relies on the routine semen analysis done as recommended by the World Health Organization (WHO). A major proportion of infertile men have normal semen parameters as per the WHO guidelines, and these account for most cases with unknown/idiopathic etiology. These cases are directly recommended to have assisted reproductive treatment rather than exploring other cost-effective and efficient andrological treatment strategies [10]. The emerging body of research highlighting vitamin D as a pleiotropic signaling molecule and its involvement in tissues and organs other than bones has encouraged studies on the role of vitamin D in human reproduction [2,11].

The presence of vitamin D receptors and metabolizing enzymes in both male and female reproductive tissues signify the role of vitamin D in human reproduction [12]. Studies have confirmed the expression of vitamin D receptors (VDRs), CYP2R1, CYP27B1, and CYP24A1 on the head and mid-piece of human sperm, elongated spermatids, epididymis, seminal vesicle, and prostate [13]. This indicates the role of vitamin D in the functions of sperm.

Most studies on the role of vitamin D deficiency have been conducted in mouse models of vitamin D deficiency. Deficiency of vitamin D has been shown to result in abnormal semen parameters such as reduced sperm concentration, motility, and a higher percentage of abnormal morphology in these mouse models of vitamin D deficiency [14]. Only a few human studies have reported an association between vitamin D deficiency and poor semen parameters [15,16]. Comprehensive studies on the roles of vitamin D and reproductive hormones in male infertility may be useful in addressing several reproductive endocrinological aspects of male fertility.

Although studies have shown a strong correlation of vitamin D deficiency with deranged semen parameters [17], there are limited studies highlighting the role of vitamin D in idiopathic male infertility, especially in Indian men. Such studies may provide potential insights into the role of vitamin D in male infertility and may result in the development of newer treatment modalities for the management of male infertility. The present study aims to evaluate vitamin D levels and reproductive hormones and their correlation with semen parameters in infertile men.

## Materials and methods

This cross-sectional study was conducted from January 2020 till December 2020 at the Indira Gandhi Institute of Medical Sciences (IGIMS), Patna, Bihar, India. Ethical clearance was obtained from the Institutional Ethics Committee, IGIMS (1223/IEC/IGIMS/2019). All participants provided a signed informed consent. To maintain confidentiality, patients were given unique laboratory identification numbers and their data were stored in a password-protected system. Only the principal investigator of the study had access to the data.

Selection of participants

In the study, 224 infertile men in the age group of 18-45 years were included. The patients were recruited from the Department of Reproductive Medicine, IGIMS, Patna, Bihar, India.

Inclusion criteria

All male partners (age: 18 to 45 years ) of the couples referred to the infertility clinic who had failed to conceive after one year of regular unprotected intercourse were included in the study.

Exclusion criteria

Couples who reported infertility due to female factors; those on vitamin D therapy, receiving testosterone or thyroxin replacement therapy, and calcium supplementation; those suffering from cancer, epilepsy, diabetes, parathyroid gland disease, hypertension, malabsorption, gastric bypass, celiac disease, inflammatory bowel disease; and those with poor general health status were excluded from the study.

Semen analysis

Semen analysis was performed strictly following the 2010 WHO guidelines. Semen samples were collected by masturbation after three to four days of abstinence. Samples were collected in sterile culture vials and left to liquefy for 20-30 minutes at room temperature. After liquefaction, the samples were analyzed for physical and morphological parameters such as volume, viscosity, liquefaction time, sperm count (million/mL), motility, and morphology (%). Sperm motility was classified as progressive, non-progressive, and immotile.

The patients were categorized as normozoospermic men (total sperm count >39 million per ejaculate, total sperm motility >40%, and normal morphology ≥4%) and those with one or more altered sperm parameter [total sperm count <39 million sperms per ejaculate, motility <40%, and normal morphology <4% (or fifth centile)].

Blood sample collection

Venous blood (2-3 mL) from the antecubital vein was collected in the morning (8 to 9 AM) from the patients. The serum was obtained by centrifugation was stored at −70°C until further analysis.

Evaluation of vitamin D levels

Vitamin D levels are not routinely checked while evaluating male patients for infertility. Therefore, the patients were explained about the objectives and their role in the study in the local language and were convinced to undergo vitamin D test. Vitamin D test was done at the institute at nominal charges which were paid by the patients. Fasting blood samples were collected from the patients to evaluate vitamin D levels. Vitamin D levels were evaluated by measuring total 25-hydroxyvitamin D in the serum by a quantitative delayed one-step competitive immunoassay using chemiluminescent microparticle immunoassay (CMIA) technology with flexible assay protocols (Architect, Abbott Diagnostics, Lake Forest, IL, USA). The patients were divided into three groups on the basis of vitamin D levels according to the Institute of Medicine guidelines [18]: Sufficient (>20 ng/mL), insufficient (12-20 ng/mL), and deficient (<12 ng/mL).

Evaluation of hormone profile

Serum follicle-stimulating hormone (FSH), luteinizing hormone (LH), and total testosterone (TT) were estimated in patients by a two-step immunoassay using CMIA technology with flexible assay protocols.

Statistical analysis

Data analysis was performed using SPSS version 21 software (IBM Corp., Armonk, NY, USA). Kolmogorov-Smirnov test was applied to test the normal distribution of the data. The continuous and categorical variables were presented as mean ± standard deviation (SD) or median (minimum, maximum) and n (%), respectively. Mann-Whitney U test was used to compare two independent groups, and the Kruskal-Wallis test was performed to compare more than two groups. Spearman’s correlation analysis was performed to investigate the association of vitamin D levels and hormone levels with semen parameters studied. Results were considered to be statistically significant if the p-value was less than 0.05.

## Results

A total of 224 infertile men were included in the study. The median age of the patients was 30 (18.45) years; median semen volume was 2 mL (0.5, 5 mL); sperm concentration was 84 million/mL (1, 180 million/mL); total percentage motility was 54% (15, 90%); progressive motility was 35% (10, 70%); normal morphology was 6% (1, 18%); vitamin D levels were 28.1 ng/mL (5.6, 46.5 ng/mL); FSH levels were 3.9 mIU/mL (0.31, 18.8 mIU/mL); LH levels were 4.8 IU/L (0.05, 12.8 IU/L, and TT levels were 789 ng/dL (165, 2,094 ng/dL). Out of the 224 patients, 124 were normozoospermic with normal semen parameters, while 100 patients had one or more altered semen parameters. There was no statistically significant difference in the age between the normozoospermic men and those with one or more semen parameters altered (p = 0.22) (Table 1). The serum vitamin D levels were significantly lower in men with one or more altered semen parameters (p < 0.0001) compared to the normozoospermic men. Men with one or more altered semen parameters had significantly higher FSH (p = 0.001) and LH levels (p < 0.0001) and significantly lower TT levels (p < 0.0001) compared to normozoospermic men (Table 1).

**Table 1:** Comparison of semen parameters, vitamin D levels, and hormone levels between normozoospermic men and those with one or more altered semen parameters Mann-Whitney test was done and the difference was considered to be significant at p < 0.05 FSH = follicle-stimulating hormone; LH = luteinizing hormone; TT = total testosterone 1 = normozoospermic men; 2 = men with one or more altered semen parameters

	Patients	N	Median (minimum, maximum)	P-Value
Age (years)	1	124	30.50 (18, 45)	0.22
	2	100	30.00 (21, 45)	
Semen volume (mL)	1	124	2 (0.50, 5.00)	0.29
	2	100	2 (0.50, 5.00)	
Sperm concentration (per mL)	1	124	90 (20, 180)	<0.0001
	2	100	76 (1, 123)	
Total motility (%)	1	124	55.50 (39, 90)	<0.0001
	2	100	40 (15, 80)	
Progressive motility (%)	1	124	40 (24, 70)	<0.0001
	2	100	20.50 (10, 70)	
Normal morphology (%)	1	124	7 (4, 13)	<0.0001
	2	100	3 (1, 18)	
FSH (mIU/mL)	1	124	3.35 (0.31, 17.8)	0.001
	2	100	4.74 (1.31, 18.79)	
LH (IU/L)	1	124	3.7 (0.05, 11.30)	<0.0001
	2	100	5.80 (0.95, 12.80)	
TT (ng/dL)	1	124	906.0 (165, 2,094)	<0.0001
	2	100	579 (400, 920)	
Vitamin D (ng/mL)	1	124	31 (7, 46.50)	<0.0001
	2	100	17.5 (5.6, 45)	

A total of 41 (18.3%) men had vitamin D levels <12 ng/mL, 41 (18.3%) had vitamin D levels between 12 and 20 ng/mL, and 142 (63.4%) had vitamin D levels >20 ng/mL. Men with serum vitamin D levels <12 ng/mL had significantly lower sperm concentration (p = 0.03), total sperm motility (p < 0.0001), progressive linear motility (p < 0.0001), and percentage normal morphology (p = 0.01) compared to men with higher serum vitamin D levels (>12 ng/mL). However, there was no significant difference in other semen parameters studied between different groups. The TT levels were significantly lower (p < 0.0001) in men with serum vitamin D levels <12 ng/mL compared to those with higher serum vitamin D levels (>12 ng/mL) (Table 2). However, there was no significant difference in the FSH and LH levels between the groups.

**Table 2:** Comparison of semen parameters and hormonal profile between patients categorized on the basis of vitamin D levels. Kruskal-Wallis test; the difference was considered significant at p < 0.05 FSH = follicle-stimulating hormone; LH = luteinizing hormone; TT = total testosterone

	<12 ng/mL (n = 41; 18.3%)	12-20 ng/mL (n = 41; 18.3%)	>20 ng/mL (n = 142; 63.4%)	P-Value
Age (years)	30 (23, 44)	30 (23, 42)	30 (18, 46)	0.31
Semen volume (mL)	2 (1, 5)	2 (0.5, 5)	2 (0.5, 5)	0.74
Sperm concentration (per mL)	78 (3, 123)	89 (7, 180)	100 (1, 180)	0.03
Total motility (%)	40 (25, 70)	52 (20, 80)	55 (15, 90)	<0.0001
Progressive motility (%)	20 (10, 40)	35 (10, 70)	40 (10, 70)	<0.0001
Normal morphology (%)	3 (1, 18)	4 (1, 13)	6 (1, 14)	0.01
FSH (mIU/mL)	4.69 (0.32, 17.49)	4.56 (0.99, 18.79)	4.49 (0.31, 17.8)	0.65
LH (IU/L)	6.30 (0.07, 12.80)	5.90 (0.06, 10.80)	5.87 (0.05, 11.6)	0.45
TT (ng/dL)	459 (165, 498)	585 (456, 991)	890 (245, 2,094)	<0.0001
Vitamin D (ng/mL)	8.2 (5.6, 9.8)	15 (12, 20)	32.75 (22, 46.5)	<0.0001

Among the normozoospermic men, six (4.8%) had vitamin D levels <12 ng/mL, 17 (13.7%) had vitamin D levels between 12 and 20 ng/mL, and 101 (81.5%) had vitamin D levels >20 ng/mL. Among men with one or more altered semen parameters, 35 (35%) had vitamin D levels <12 ng/mL, 24 (24%) had vitamin D levels between 12 and 20 ng/mL, and 41 (41%) had vitamin D levels >20 ng/mL.

The serum TT levels were significantly lower in patients with serum vitamin D levels <12 ng/mL in both normozoospermic men and those with one or more altered semen parameters compared to patients with higher vitamin D levels. There was no significant difference in other sex hormones. The sperm concentration, total motility, linear progressive motility, and percentage normal morphology were significantly lower in patients with serum vitamin D levels <12 ng/mL in both the normozoospermic men and those with one or more altered semen parameters compared to patients with higher vitamin D levels (Table 3).

**Table 3:** Comparison of different parameters between patients grouped according to different vitamin D levels in normozoospermic men and those with one or more altered semen parameters. Kruskal-Wallis test; the difference was considered significant at p < 0.05 FSH = follicle-stimulating hormone; LH = luteinizing hormone; TT = total testosterone

Test statistics										
Patients		Age	Volume	Sperm concentration	% Total motility	% Progressive motility	% normal Morphology	FSH	LH	TT
Normozoospermic men	Chi-square	0.247	6.841	13.34	20.34	17.88	19.67	1.278	1.28	25.283
	P-value	0.884	0.687	0.03	0.02	0.01	0.04	0.53	0.53	0.000
Men with one or more altered semen parameters	Chi-square	3.367	2.608	14.87	21.89	15.891	15.65	3.78	3.78	86.603
	P-value	0.186	0.271	0.01	0.03	<0.0001	0.02	0.15	0.15	<0.0001

Spearman’s correlation analysis was done in both normozoospermic and those with one or more abnormal semen parameters. It was observed that semen parameters such as sperm concentration, total motility, linear progressive motility, and morphology positively correlated with the levels of serum vitamin D and TT (Table 4).

**Table 4:** Spearman’s correlation among patients with normal and abnormal semen parameters. r = correlation coefficient. r was considered significant at p < 0.05 **Correlation is significant at the 0.01 level (two-tailed); *Correlation is significant at the 0.05 level (two-tailed) FSH = follicle-stimulating hormone; LH = luteinizing hormone; TT = total testosterone

Normozoospermic men								
		Age	Volume	Sperm concentration		Total motility	Progressive motility	Normal morphology
FSH	r	-0.13	0.09	-0.07		0.07	0.18^*^	0.004
	p	0.14	0.31	0.47		0.47	0.05	0.97
LH	r	0.11	0.17	0.02		0.02	0.04	-0.06
	p	0.23	0.06	0.79		0.84	0.66	0.51
TT	r	0.06	0.06	0.38^**^		0.45^**^	0.44^**^	0.19^*^
	p	0.52	0.52	0.01		0.04	0.02	0.03
Vitamin D	r	0.16	-0.15	0.45		0.54	0.46	0.31
	p	0.08	0.09	0.01		0.02	0.01	0.03
Men with one or more altered semen parameters								
FSH	r		-0.16	-0.02	0.02^*^	-0.01	0.01	0.12
	p		0.10	0.80	0.07	0.91	0.93	0.16
LH	r		0.03	0.12	0.04	-0.16	-0.14	0.15
	p		0.81	0.23	0.69	0.12	0.18	0.13
TT	r		0.19	0.15	0.47^*^	0.55^**^	0.34^**^	0.22^*^
	p		0.06	0.13	0.02	0.01	0.001	0.03
Vitamin D	r		0.17	0.19	0.61	0.31^*^	0.38^**^	0.24^*^
	p		0.11	0.06	0.001	0.03	0.000	0.02

## Discussion

In the present cross-sectional study involving infertile men (normozoospermic and those with one or more altered semen parameters), it was observed that men with one or more semen abnormalities had significantly lower vitamin D levels, higher FSH and LH levels, and lower serum TT levels compared to infertile men with normal semen parameters. Patients with lower vitamin D levels (<12 ng/mL) had significantly lower sperm count, motility (both total and linear progressive), normal morphology, and lower TT levels compared to patients with higher serum vitamin D levels (>12 ng/mL). This trend was seen in both patients with normal semen parameters (normozoospermic) as well as in those with one or more altered semen parameters. Serum vitamin D levels and TT levels were positively correlated with semen parameters studied (concentration, motility, and morphology).

About 10 to 15% of couples are infertile in the industrialized countries worldwide, and in approximately 50% of these cases, there is involvement of male factors [19]. Abnormal semen parameters are important contributing factors of infertility in about half of infertile couples. However, there are 30 to 40% of infertile couples in which male-related factors are absent. In idiopathic cases of male infertility where semen parameters are normal, several other factors such as oxidative stress, endocrinal factors, and genetic abnormalities may be the cause of infertility. According to the WHO, the prevalence of infertility in India varies from 3.9 to 16.8% among different states [20].

Recently, vitamin D has been given more attention by researchers due to its multitude of functions targeting several organs and systems such as the intestine, skeletal system, kidneys, and parathyroid glands [21]. The principal function of vitamin D is to promote bone mineralization by regulating calcium and phosphorus homeostasis. In addition to this, recently it has been shown that vitamin D also targets organs and systems such as adipose tissue, thyroid, immune system, pancreas, cardiovascular system, central nervous system, and the reproductive system. Vitamin D deficiency has been implicated in a spectrum of clinical conditions including hyperparathyroidism, rickets, osteomalacia, obesity, thyroid dysfunction, autoimmune diseases, diabetes mellitus, cardiovascular diseases, dementia, and cancer [22].

Ergocalciferol (vitamin D2) and cholecalciferol (vitamin D3) are two biologically important forms of vitamin D. Vitamin D3 is mainly synthesized in the skin. However, it is not biologically active. 1-α,25-dihydroxyvitamin D3 which is the biologically active form of vitamin D3 is produced via a two-step process. First, the inactive form of vitamin D is converted into 25-hydroxyvitamin D3 by a hepatic enzyme, which is then converted into 1-α,25-dihydroxyvitamin D3 by a renal enzyme. VDRs present on the target organs and tissues facilitate the binding of vitamin D and execute their biological activities. Expression of VDRs in various components of the male reproductive system such as prostate, testis, ejaculated spermatozoa, and Sertoli cells are suggestive of their role in male fertility and reproduction [12,13]. The presence of vitamin D metabolizing enzymes in the human testis, ejaculatory tract, and mature spermatozoa indicates the important role of vitamin D in the process of spermatogenesis [12,13]. Moreover, studies have shown that hypovitaminosis D, in both animals and humans, is associated with poor semen and hormone functions [22].

Studies have also proposed that vitamin D may have important roles in Ca^2+^-dependent processes such as hyperactivated motility, capacitation, and acrosome reaction. The role of vitamin D in sperm survival and motility has also been shown. Vitamin D regulates sperm survival and motility by modulating important processes such as cholesterol efflux and phosphorylation of tyrosine and threonine residues on specific proteins [13]. Although several studies have evaluated the role of vitamin D in male infertility, there is no consensus established to date on the role of vitamin D in semen quality and function. Therefore this study was conducted to evaluate the role of serum levels of vitamin D and hormones (FSH, LH, and TT) on semen parameters in infertile men with normal and abnormal semen parameters. A recent study highlighted no significant association of serum vitamin D levels with semen parameters. There was no significant difference in serum vitamin D levels between normozoospermic and oligoasthenoteratozoospermic men. However, serum vitamin D levels were found to be positively correlated with sperm motility in all patients and with normal morphology in normozoospermic men [23].

Several studies have been conducted to evaluate the effect of hypovitaminosis D on semen parameters and the fertility status of men. Many studies have reported a positive correlation between serum vitamin D levels and sperm concentration, total and linear progressive motility, and morphology [24,25]. A recent study showed that men with vitamin D deficiency had significantly lower total and progressive sperm motility, suggesting that vitamin D deficiency may affect semen quality in infertile men [26].

Consistent with the findings of these studies, in the present study, we found a strong positive association of serum vitamin D levels on both sperm total and linear progressive motility. We also observed a strong positive association of vitamin D levels with sperm concentration and morphology. Men with vitamin D levels below 12 ng/mL had significantly lower sperm concentration and normal morphology compared to those with higher serum vitamin D levels.

Despite these reports, there is no unanimous agreement on the association of vitamin D deficiency with poor semen parameters. According to a systematic review conducted by Cito et al. [22], several studies have shown no correlation between serum levels of vitamin D and sperm count and morphology. While there are several studies that indicated a positive correlation between serum vitamin D levels and sperm count, most of these studies highlighted that vitamin D status is not related to sperm morphology. However, there are a few studies that showed a positive association of serum vitamin D levels with sperm morphology [22].

Interventional studies conducted on oligoasthenozoospermic men to evaluate the effects of vitamin D supplementation on semen parameters have shown that supplementation with vitamin D increased sperm progressive motility and pregnancy rates in men supplemented with vitamin D compared to those who were not supplemented [27]. However, another study showed that vitamin D supplementation did not affect semen parameters in oligoasthenozoospermic men, but showed that the rate of spontaneous pregnancies and chance for a live birth were increased compared with the placebo group [28].

The difference in observations in different studies may be due to heterogeneity in study design, methodology used, patient type, age group of patients, and different cut-off values considered categorizing patients as vitamin D deficient.

There is inconsistency in the literature regarding the role of vitamin D in the production of sex hormones. A few studies have shown a positive correlation of serum vitamin D levels with total TT [14]. Another study reported a weak relationship of vitamin D levels with sex hormones in men [29]. There are several other studies that have found no association of serum vitamin D levels with sex hormones in men [30]. Consistent with these findings, we also observed no association of serum vitamin D levels with sex hormones such as FSH and LH. However, we observed that the serum TT levels were significantly lower in the group of infertile patients with vitamin D levels <12 ng/mL and were highest in the group of patients with vitamin D levels <20 ng/mL. This indicates an association of vitamin D levels with TT. However, in contrast to the current findings, a few studies have reported no association of serum TT levels with vitamin D levels [23]. In agreement with our findings, a previous study showed a positive association of vitamin D levels with TT levels in men [30]. Another study that also segregated infertile men into normozoospermic men and those with one or more altered semen parameters reported that serum vitamin D levels were higher in normozoospermic men compared to those with one or more altered semen parameters. They also showed a positive association of vitamin D levels with semen parameters studied such as concentration, motility, and morphology [24].

It was observed in the present study that, although the serum levels of FSH and LH did not correlate with semen parameters except for TT, which was positively correlated with sperm concentration, motility, and morphology in both normozoospermic men and those with one or more abnormal semen parameters, normozoospermic men had significantly lower FSH and LH levels and higher TT levels compared to those with one or more abnormal semen parameters. We observed that TT correlated well with vitamin D levels and semen parameters. Therefore, the effects of vitamin D on the semen parameters may be mediated by regulation of TT levels by vitamin D. This hypothesis needs further confirmation.

The major limitation of the current study was the absence of a control group. However, the present cohort has two different groups of infertile men: those with normal semen parameters and those with one or more abnormal semen parameters. Despite the absence of a fertile control group, the present study substantiates the association of serum vitamin D levels with semen parameters in infertile men. Furthermore, the present study indicates and verifies the association of TT levels with semen parameters in infertile men. The findings of the present study warrant further extensive studies with larger cohorts of patients to confirm the association of TT levels with hypovitaminosis D and semen parameters.

## Conclusions

Infertile men have vitamin D deficiency and further studies are needed to understand the effect of vitamin D supplementation on semen parameters.

## References

[1] Fields J, Trivedi NJ, Horton E, Mechanick JI (2011). Vitamin D in the Persian Gulf: integrative physiology and socioeconomic factors. Curr Osteoporos Rep.

[2] Anderson PH, Turner AG, Morris HA (2012). Vitamin D actions to regulate calcium and skeletal homeostasis. Clin Biochem.

[3] Savastano S, Barrea L, Savanelli MC, Nappi F, Di Somma C, Orio F, Colao A (2017). Low vitamin D status and obesity: role of nutritionist. Rev Endocr Metab Disord.

[4] Nettore IC, Albano L, Ungaro P, Colao A, Macchia PE (2017). Sunshine vitamin and thyroid. Rev Endocr Metab Disord.

[5] Altieri B, Grant WB, Della Casa S (2017). Vitamin D and pancreas: the role of sunshine vitamin in the pathogenesis of diabetes mellitus and pancreatic cancer. Crit Rev Food Sci Nutr.

[6] Muscogiuri G, Altieri B, de Angelis C, Palomba S, Pivonello R, Colao A, Orio F (2017). Shedding new light on female fertility: the role of vitamin D. Rev Endocr Metab Disord.

[7] Föcker M, Antel J, Ring S (2017). Vitamin D and mental health in children and adolescents. Eur Child Adolesc Psychiatry.

[8] Tirabassi G, Cutini M, Muscogiuri G (2017). Association between vitamin D and sperm parameters: clinical evidence. Endocrine.

[9] G R, Gupta A (2014). Vitamin D deficiency in India: prevalence, causalities and interventions. Nutrients.

[10] Hallak J (2017). Utility of sperm DNA fragmentation testing in different clinical scenarios of male reproductive abnormalities and its influence in natural and assisted reproduction. Transl Androl Urol.

[11] Foresta C, Strapazzon G, De Toni L (2011). Bone mineral density and testicular failure: evidence for a role of vitamin D 25-hydroxylase in human testis. J Clin Endocrinol Metab.

[12] Blomberg Jensen M, Nielsen JE, Jørgensen A (2010). Vitamin D receptor and vitamin D metabolizing enzymes are expressed in the human male reproductive tract. Hum Reprod.

[13] Aquila S, Guido C, Perrotta I, Tripepi S, Nastro A, Andò S (2008). Human sperm anatomy: ultrastructural localization of 1alpha,25-dihydroxyvitamin D receptor and its possible role in the human male gamete. J Anat.

[14] Wehr E, Pilz S, Boehm BO, März W, Obermayer-Pietsch B (2010). Association of vitamin D status with serum androgen levels in men. Clin Endocrinol (Oxf).

[15] Lerchbaum E, Pilz S, Trummer C, Rabe T, Schenk M, Heijboer AC, Obermayer-Pietsch B (2014). Serum vitamin D levels and hypogonadism in men. Andrology.

[16] Sun W, Chen L, Zhang W, Wang R, Goltzman D, Miao D (2015). Active vitamin D deficiency mediated by extracellular calcium and phosphorus results in male infertility in young mice. Am J Physiol Endocrinol Metab.

[17] de Angelis C, Galdiero M, Pivonello C (2017). The role of vitamin D in male fertility: a focus on the testis. Rev Endocr Metab Disord.

[18] Rosen CJ, Abrams SA, Aloia JF (2012). IOM committee members respond to Endocrine Society vitamin D guideline. J Clin Endocrinol Metab.

[19] Aston KI, Krausz C, Laface I, Ruiz-Castané E, Carrell DT (2010). Evaluation of 172 candidate polymorphisms for association with oligozoospermia or azoospermia in a large cohort of men of European descent. Hum Reprod.

[20] (2021). World Health Organization. Infecundity, infertility, and childlessness in developing countries. DHS Comparative Reports No 9. https://www.who.int/reproductivehealth/publications/infertility/DHS_9/en/.

[21] Dusilová-Sulková S (2009). Vitamin D metabolism and vitamin D traditional and nontraditional, target organs: implications for kidney patients. J Ren Care.

[22] Cito G, Cocci A, Micelli E (2020). Vitamin D and male fertility: an updated review. World J Mens Health.

[23] Azizi E, Naji M, Shabani-Nashtaei M, Aligholi A, Najafi A, Amidi F (2018). Association of serum content of 25-hydroxy vitamin D with semen quality in normozoospermic and oligoasthenoteratozoospermic men. Int J Reprod Biomed.

[24] Rehman R, Lalani S, Baig M, Nizami I, Rana Z, Gazzaz ZJ (2018). Association between vitamin D, reproductive hormones and sperm parameters in infertile male subjects. Front Endocrinol (Lausanne).

[25] Akhavizadegan H, Karbakhsh M (2017). Comparison of serum vitamin D between fertile and infertile men in a vitamin D deficient endemic area: a case-control study. Urologia.

[26] Blomberg Jensen M, Gerner Lawaetz J, Andersson AM (2016). Vitamin D deficiency and low ionized calcium are linked with semen quality and sex steroid levels in infertile men. Hum Reprod.

[27] Deng X, Song Y, Manson JE (2013). Magnesium, vitamin D status and mortality: results from US National Health and Nutrition Examination Survey (NHANES) 2001 to 2006 and NHANES III. BMC Med.

[28] Blomberg Jensen M, Lawaetz JG, Petersen JH, Juul A, Jørgensen N (2018). Effects of vitamin D supplementation on semen quality, reproductive hormones, and live birth rate: a randomized clinical trial. J Clin Endocrinol Metab.

[29] Wulaningsih W, Van Hemelrijck M, Michaelsson K (2014). Association of serum inorganic phosphate with sex steroid hormones and vitamin D in a nationally representative sample of men. Andrology.

[30] Anic GM, Albanes D, Rohrmann S (2016). Association between serum 25-hydroxyvitamin D and serum sex steroid hormones among men in NHANES. Clin Endocrinol (Oxf).

